# Extracellular vesicle therapy for traumatic central nervous system disorders

**DOI:** 10.1186/s13287-022-03106-5

**Published:** 2022-09-02

**Authors:** Jing Zhang, Weipeng Shi, Di Qu, Tengbo Yu, Chao Qi, Haitao Fu

**Affiliations:** 1 Department of Sports Medicine, The Affiliated Hospital of Qingdao University, Qingdao University, Qingdao, 266071 China; 2 Medical Department of Qingdao University, Qingdao, China

**Keywords:** Traumatic CNS disorder, Extracellular vesicles, Biomarkers, Drug delivery

## Abstract

Traumatic central nervous system (CNS) disorders have catastrophic effects on patients, and, currently, there is no effective clinical treatment. Cell transplantation is a common treatment for traumatic CNS injury in animals. In recent years, an increasing number of studies have reported that the beneficial effect of transplanted cells for CNS repair is mediated primarily through the extracellular vesicles (EVs) secreted by the cells, in which microRNAs play a major role. Accordingly, numerous studies have evaluated the roles and applications of EVs secreted by different cell types in neurological diseases. Furthermore, due to their unique biological features, EVs are used as disease biomarkers and drug delivery systems for disease prevention and treatment. We discuss current knowledge related to EVs, focusing on the mechanism underlying their effects on traumatic CNS diseases, and summarize existing research on the potential clinical utility of EVs as disease biomarkers and drug delivery systems.

## Introduction

Traumatic central nervous system (CNS) disorders are caused by a variety of events, the most common of which are traumatic brain injury (TBI) and spinal cord injury (SCI). Following trauma to the brain or spinal cord, patients frequently develop varying degrees of impairment. Although most patients with CNS injury experience spontaneous functional recovery, it is often modest and limited. Due to the complex pathological mechanisms of CNS injury, no effective clinical treatments are available.

Significant progress has been made in the use of cell transplantation to investigate the treatment of CNS diseases. Early clinical trials have reported the effectiveness of cell transplantation as an alternative treatment of CNS diseases, but its feasibility and long-term effects are largely unknown [1–3]. The clinical application of cell transplantation is limited by tumorigenesis, low survival rates, and immune rejection [4]. Therefore, identifying mediators that promote the interaction between transplanted and host cells, while preventing the complications of cell transplantation, is necessary for the clinical treatment of traumatic CNS diseases. Previous studies have suggested that the efficacy of mesenchymal stem cell (MSC) therapy is attributable to paracrine-mediated mechanisms rather than the engraftment and differentiation of cells at the injury site [5, 6]. The paracrine effects of MSCs are mediated by the secreted products, known as extracellular vesicles (EVs), which have recently gained significant attention [7, 8]. EVs have significant therapeutic potential due to their abundant cargo and their ability to deliver it to recipient cells, which act on multiple signaling pathways [9–11]. The cytoprotective, angiogenic, and regenerative effects of MSCs can be reproduced by the administration of EVs released by cells while avoiding the risk factors associated with MSC administration [11–13]. In a preclinical experiment, the motor and cognitive functions of TBI rats were significantly improved after injection of EVs derived from hypoxia-preconditioned bone marrow-derived MSCs (BMSCs) into the tail vein, showing a therapeutic effect superior to that of MSCs [14].

A large number of studies have reported positive effects of EVs in the treatment of neurological diseases, and this treatment method has more advantages than cell transplantation. For example, as nanoscale lipid membrane vesicles, EVs can cross the blood–brain barrier/blood–spinal cord barrier (BBB/BSCB) without destroying their integrity [15]. EVs can be conveniently delivered through intravenous or intranasal delivery [16, 17]. In addition, the biologically active molecules inside EVs, especially microRNAs (miRNAs) and proteins, allow EVs to be used as biomarkers for early diagnosis and determination of disease progression and treatment response [18]. EVs are also used as carriers to deliver ncRNAs, cytokines, and traditional Chinese medicines and can therefore be applied in nanotherapeutics [19]. The membrane protein on the EV surface can be engineered to target the injury site, improve treatment specificity, and increase treatment efficiency [20]. Therefore, EVs are expected to become a feasible treatment option for the treatment of CNS injury and replace cell transplantation.

In the present review, we discuss the important role of EVs in the CNS, highlighting the potential mechanisms by which EVs promote functional recovery following CNS injury. We also discuss the current applications of EVs as biomarkers and drug carriers in neurological diseases and provide evidence that modified EVs affect functional recovery after injury.

## EVs

**Fig. 1:**
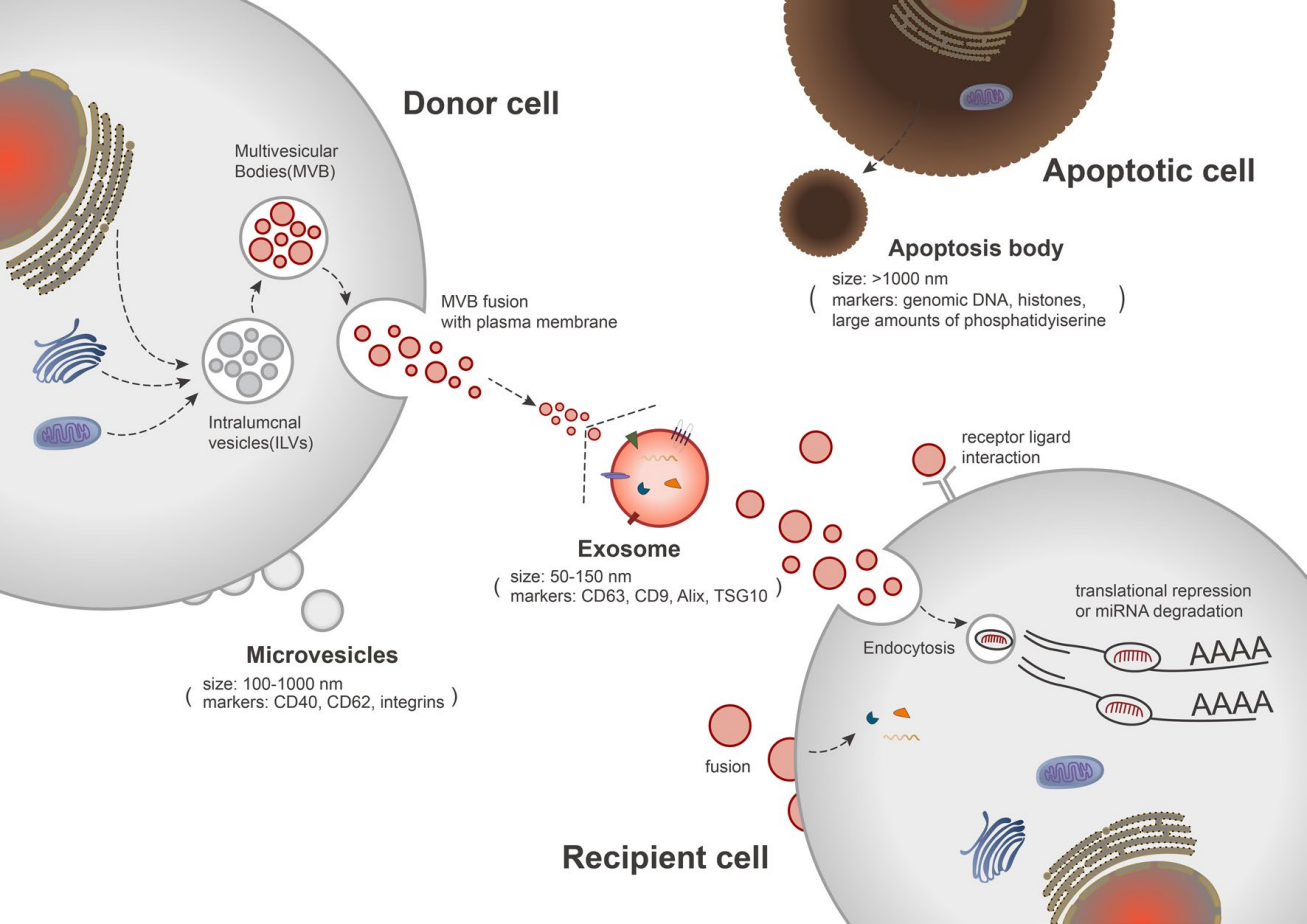
Biogenesis, characterization, and uptake of vesicle subtypes. The early endosomal membrane is depressed to form intraluminal vesicles, which aggregate to generate intracellular multivesicular bodies (MVBs). In turn, the MVBs isolate specific proteins, lipids, and other components in the cytoplasm and then generate exosomes (50–150 nm) via fusion with the plasma membrane or transport lysosomes for degradation. The released exosomes can enter the recipient cells via three routes: ligand–receptor binding on the receptor cell surface, endocytosis, and membrane fusion. Other types of vesicles include microvesicles and apoptotic bodies. Microvesicles (100–1000 nm) are released directly from living cells; they bud from the plasma membrane, are released into the extracellular environment after cell activation, and enter recipient cells through vesicle fusion or endocytosis. Meanwhile, apoptotic bodies (> 1000 nm) are released from dead cells without direct fusion to the plasma membrane

EVs are nanoscale membrane vesicles released by almost all cell types, including exosomes, microvesicles, and apoptotic bodies [21, 22]. These vesicle subtypes involve different biogenesis pathways and are often differentiated by their size, surface proteins, and contents (Fig. 1). Because of the lack of a standardized, uniform separation method for characterization, several terms are often used interchangeably to describe EVs and exosomes. Therefore, we used the terms EVs and exosomes interchangeably in this paper.

One reason for the significant interest in EVs is the abundance of their cargos, which contain cytokines, lipids, proteins, and nucleic acids, such as DNA, noncoding RNA, ribosomal RNA (rRNA), and miRNA. EVs derived from different cell types differ in terms of their contents, including transcriptomes, proteomes, and lipidomes [23–25]. These vesicles mediate cell signaling by delivering their cargo to recipient cells, consequently leading to the expression of target genes [26]. EVs have similar functions to their source cells and may vary according to the status of the source cell. In vitro studies have reported that the use of MSC-EVs in the acute phase after SCI can significantly reduce the expression of pro-inflammatory cytokines in the spinal cord parenchyma in the early stage of secondary injury, indicating that MSC-EVs are as effective as their parent cells [27]. In addition, EVs released by the same cells at different stages may have different functions. For example, MSCs secrete exosomes with extracellular matrix mineralization properties only at an early stage of osteogenic differentiation [28]. Thus, EV composition is a tightly regulated process that may be affected by environmental factors, including cell activation and stress conditions.

Previous studies have shown that EVs exert multifactorial therapeutic effects by delivering several genetic substances to receptor cells that alter their physiological functions. MiRNAs are important functional regulators of receptor cells involved in the pathogenesis and recovery of several neurological diseases. A previous study isolated EVs from differentiated PC12 cells and MSCs and administered the EVs intravenously to SCI rats. The results showed that the relative expression levels of miR19b and miR21 were increased in MSC-EVs, which inhibited the activation of PTEN [29]. Therefore, MSC-EVs play an active role in promoting neuron generation and functional recovery after SCI by regulating miR19b and miR21. In addition, miR-199a-3p/145-5p, which is relatively highly expressed in exosomes from human umbilical cord MSCs (hUC-MSCs), can be transferred into the neurons of SCI rats to promote neurite outgrowth and angiogenesis [30]. Therefore, EVs play a role in various biological mechanisms by delivering specific mRNAs or miRNAs.

The BBB/BSCB separates the brain from the peripheral circulation to maintain homeostasis. From a therapeutic perspective, this barrier restricts the access of therapeutic cells and drugs to target sites in the brain parenchyma, resulting in inadequate efficacy in CNS diseases [31]. Exosomes cross the BBB from blood vessels into neural tissue and establish a communication pathway across the CNS [32]. In a previous study, intranasally administered curcumin-loaded EVs crossed the BBB and localized in brain microglia, resulting in functional recovery of experimentally induced immune encephalomyelitis mice [33]. Another study showed that exosomes can carry cargos, such as miR-193b-3p, across the BBB to the injury site after subarachnoid hemorrhage to alleviate neuroinflammation in the hemorrhage area, suggesting that exosomes can still cross the BBB under stressful conditions [34]. The properties and functions of EVs reported previously suggest their significant potential for clinical use, thereby providing a new strategy for the treatment of neurological diseases.

## EVs in CNS

**Fig. 2:**
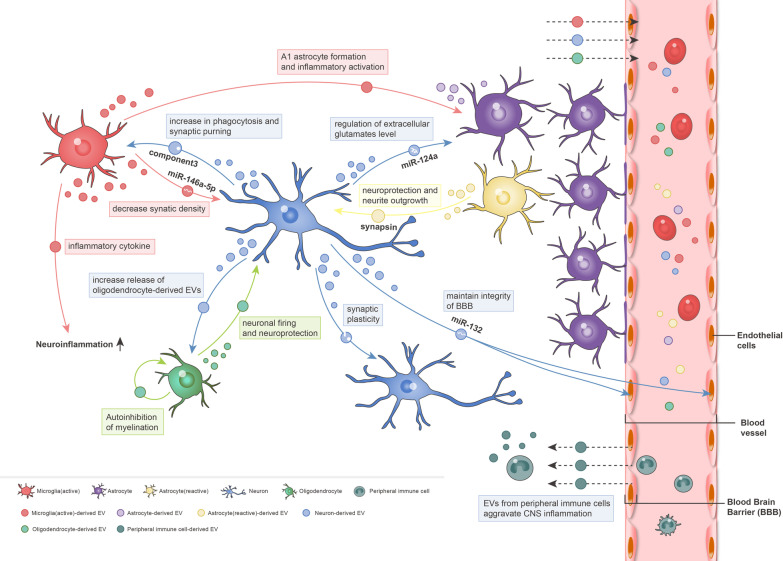
Roles of extracellular vesicles (EVs) in the central nervous system (CNS) after insult. Almost all CNS cells can secrete and uptake EVs containing various bioactive molecules, which affect receptor cell function under various physiological and pathological conditions. Neurons can release EVs to other neurons, thereby modulating synaptic plasticity in the CNS. Activated microglia/EV complexes containing miR-146a-5p can be internalized by neurons, in which they reduce the synaptic density under inflammatory conditions. Moreover, neuronal EVs can upregulate the expression of complement component 3 in microglia, thereby enhancing its phagocytic and synaptic pruning abilities under both developmental and pathological conditions. Neuronal EVs containing miR-124a act on astrocytes to regulate extracellular glutamate concentrations. Activated astrocytes release EVs carrying synaptophysin, which acts on neurons to promote neuronal growth under conditions of high neuronal activity and/or oxidative stress. Furthermore, neuronal exosomal miR-132 interacts with endothelial cells, which maintained the integrity of the blood–brain barrier in an intact zebrafish larvae model. Polarization of neurons can prompt oligodendrocytes to release more EVs, which enhanced neuronal tolerance and firing frequency in a model of cerebral ischemia. EVs also act on oligodendrocytes, thereby inhibiting their differentiation and myelination. In addition, glial cells in the CNS and immune cells in the peripheral nervous system can modulate the inflammatory response after nerve injury through extensive communication mediated by EVs

The construction and maintenance of various neuronal circuits in the CNS require complex and coordinated communication between various types of nerve cells. EVs can be released from different cell types, including neurons [35], oligodendrocytes [36], astrocytes [37, 38], and microglia [39]; these EVs participate in the signal shuttle between glial cells and neurons as intercellular communication mediators, promoting synaptic assembly and regulating neurological activities and the cell immune response [40, 41](Fig. 2).

EVs released by neurons can affect the function of recipient cells. EVs released by neurons can be captured by other neurons and regulate synaptic plasticity in a synaptic activity-dependent manner [42–44]. Exosomal miR-124a obtained from mouse primary cortical neurons can be internalized by astrocytes and increases the expression of the glial glutamate transporter EAAT2/GLT, resulting in changes in extracellular glutamate levels [45]. Furthermore, EVs released from rat cortical neurons reduced lipopolysaccharide (LPS)-induced microglia pro-inflammatory responses and promoted anti-inflammatory responses [46]. Neuronal exosomes can upregulate the expression level of complement component 3 in microglia, to stimulate glial phagocytosis and enhance synaptic pruning during neuronal remodeling [47]. These findings suggest that neurons mediate communication with microglia via EVs to regulate microglial activity. In addition, neuronal exosomes containing miR-132 can directly interact with endothelial cells to regulate BBB integrity [48]. Disruption of neuronal miR-132 expression or exosome secretion impairs the integrity of the brain vasculature.

Glial cell-derived EVs can regulate the viability and function of neurons and other cells. Under conditions of high neuronal activity/oxidative stress, glial cells interact with neurons by releasing exosomes containing synapsin, to promote neurite growth and neuronal survival [49]. The release of oligodendrocyte exosomes can be increased by the enhanced glutamate activity induced by potassium-induced neuronal depolarization. These oligodendrocyte-derived exosomes can be internalized by neurons, thereby enhancing neuronal stress tolerance and firing rates [50, 51]. Furthermore, oligodendrocyte-derived vesicles enter the extracellular space, where they inhibit oligodendrocyte differentiation and myelin formation. The autoinhibitory effect of EVs released from oligodendrocytes can be reduced by several factors in a neuronal conditioned medium, which indicates oligodendrocyte–neuron communication via EVs [52]. Microglia release vesicles containing pro-inflammatory cytokines in the presence of ATP released by astrocytes or dying cells at injury sites; these vesicles participate in the inflammatory response, which triggers the acute inflammatory response [37, 39]. In in vitro culture, neuronal internalization of microglia-derived EVs containing miR-146a-5p decreased the synaptic density of cultured neurons [53]. Astrocyte-derived exosomal prion protein (PrP) enters the neurons under ischemia–hypoxia and enhances neuronal survival [54]. Because of the location and biological properties of astrocytes, the end-feet of individual astrocytes are often in contact with the BBB, possibly mediating neuronal-to-peripheral blood communication through exosomes [55].

EVs mediate communication between the CNS and periphery. After CNS insult, microglia- or astrocyte-derived inflammatory EVs enter the peripheral circulation, where they stimulate inflammatory responses in peripheral immune cells [56, 57]. Furthermore, EVs derived from the peripheral immune cells infiltrate the CNS inflammatory site, where they exacerbate the response to injury as neuroinflammatory mediators [58]. An in vivo study reported the contribution of peripheral circulating inflammatory EVs to neuroinflammation when purified circulating EVs from the sera of LPS-challenged mice were intravenously injected into normal adult mice. The recipient mice exhibited increased microglial activation and systemic pro-inflammatory cytokine production. Moreover, the intravenously injected EVs are mainly taken up by microglia, implying that peripheral monocyte–macrophage lineage cells may be the main source of the potent EVs. This is consistent with the common assumption that the LPS response is mediated by myeloid cells. A recent study showed that EVs secreted by the LPS-stimulated macrophage RAW264.7 cell line can enhance the polarization of microglia from the M1 to M2 phenotype in vitro and in vivo, thereby inhibiting the inflammatory response, exerting a neuroprotective effect in ischemic stroke, and effectively promoting functional recovery [59].

As mentioned previously, EVs secreted by different cells establish a highly regulated and complex network within the nervous system (Fig. 2). Unlike the widely disseminated source cells, EVs show distinct tissue/cell homing rather than randomly interacting with nearby recipient cells [60]. Determining the cellular communication mediated by EVs secreted from different cells will help us to understand the nature of biological signals, explore new uses of EVs in the treatment of neurological diseases, and develop new therapeutic strategies.

## Mechanisms underlying the effects of EVs in traumatic CNS injury repair

**Fig. 3:**
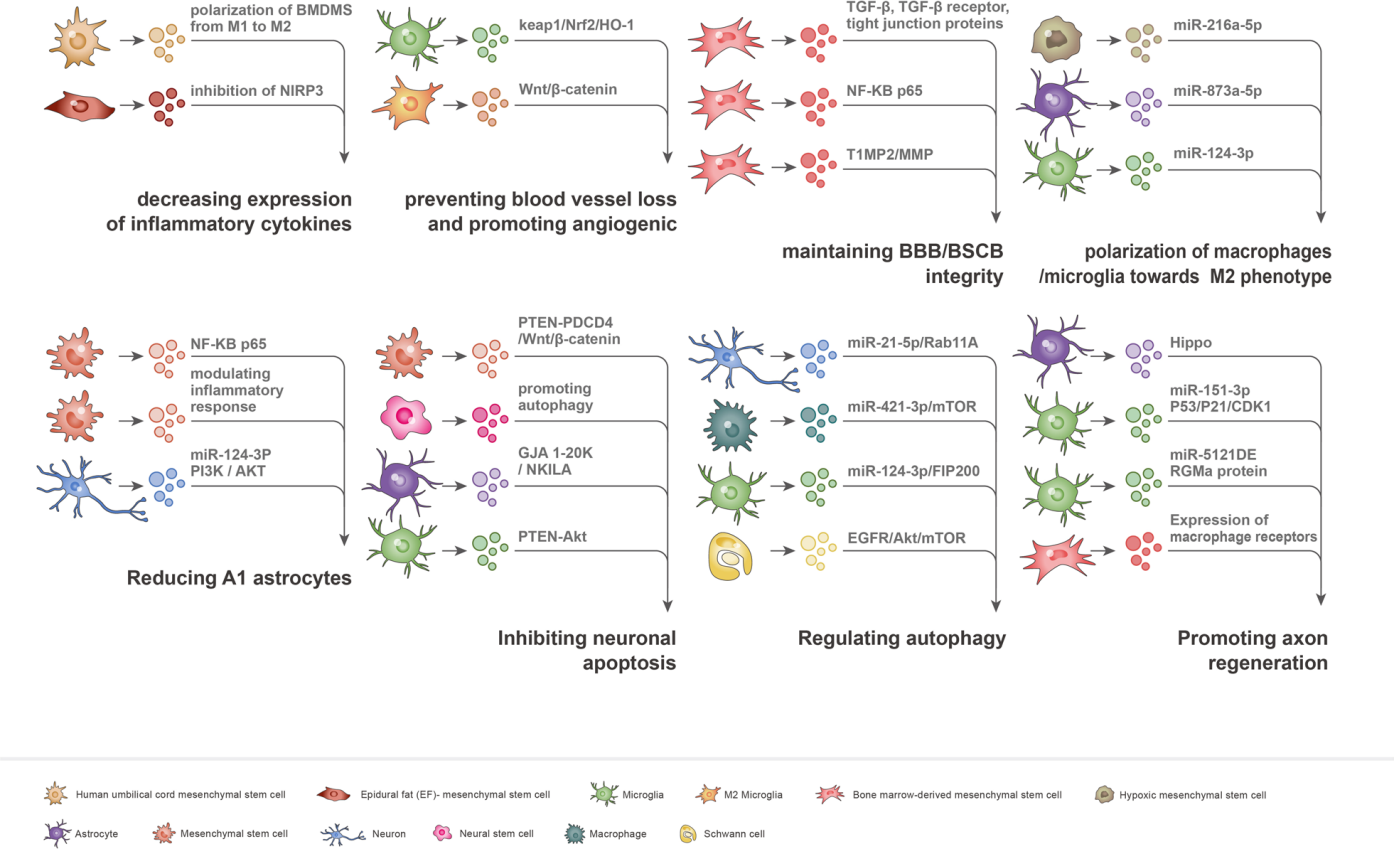
Mechanisms underlying the reparative effects of extracellular vesicles (EVs) in traumatic central nervous system (CNS) injury. The main mechanisms are as follows: inflammatory regulation, angiogenesis, maintaining of BBB/BSCB integrity, polarization of microglia toward M2 phenotype, suppression of A1 astrocyte expression, inhibition of apoptosis, regulation of autophagy, and axon regeneration. EVs from a variety of cell types restore function after CNS by modulating the bioactive molecules (e.g., RNA, proteins, and active bodies) mediating various signaling pathways

EVs participate in CNS repair through a variety of mechanisms, including inflammatory responses, angiogenic activity, the modulation of BBB/BSCB integrity, polarization of microglia, suppression of A1 astrocyte expression, apoptosis, autophagy, and axon regeneration (Fig. 3). In addition to discussing how these mechanisms promote CNS repair, this study also provides definitions.

### Regulation of inflammatory responses

#### Inhibition of inflammatory responses

The inflammatory response induced by CNS injury is the earliest and most important initiating event for the secondary injury process, which often hinders functional recovery [61]. Inhibition of the inflammatory responses in the early stages of CNS injury may be an effective method of neuroprotection. Studies have shown that hUC-MSC-derived exosomes attenuate inflammation in the injured area and promote the healing of SCI by downregulating inflammatory cytokines, such as TNF-α, MIP-1α, IL-6, and IFN-γ. This process may be achieved by efficiently triggering the polarization of bone marrow-derived macrophages (BMDMs) from the M1 to M2 phenotype [62]. Recently, the nucleotide-binding domain-like receptor protein 3 (NLRP3) inflammasome has been shown to play a key role in the secondary injury phase of SCI. Systemic administration of EVs derived from epidural fat (EF)-MSCs to the SCI model significantly inhibited the activation of the NLRP3 inflammasome and decreased the expression of inflammatory cytokines after SCI [63].

#### Promoting the polarization of macrophages/microglia toward the M2 phenotype

The phenotype of macrophage/microglia polarization determines the effects on inflammation, and induction of cell polarization to the M2 type accelerates functional recovery. Exosomes derived from M2 macrophages promote the M2 polarization of macrophages with the help of a putative miRNA–mRNA network and provide beneficial active molecules for SCI repair [64]. Treatment of SCI rats with hypoxic MSC-derived exosomes shifted microglial polarization from the M1 to M2 phenotype in vivo and in vitro and promoted functional behavioral recovery, which involved hypoxic exosomal miR-216a-5p [65]. Astrocyte-derived exosomes enriched with miR-873a-5p can promote the polarization of microglia from the M1 to M2 phenotype by inhibiting ERK and NF-κB signaling pathways, attenuate microglia-induced neuroinflammation, and improve neurological deficits after TBI [66]. Microglial exosomes also inhibit neuroinflammation by promoting microglia polarization to the M2 type, in which miR-124-3p plays a key role [67].

#### Reducing A1 astrocytes

The activation of A1 astrocytes has neurotoxic effects on myelin, synapses, and neurons; therefore, inhibiting A1 astrocytes has therapeutic potential for nervous system damage. The administration of BMSC-EVs can effectively inhibit the activation of A1 neurotoxicity-responsive astrocytes and improve recovery after traumatic SCI. The reduction in A1 glial cell activation successfully inhibits glial scarring, suppresses the inflammatory process, and promotes the regeneration of blood vessels and nerves [68]. Neurotoxic A1 astrocytes are regulated by the NF-κB pathway [69]. MSC-derived exosomes can effectively reduce the A1 astrocytes induced after SCI by inhibiting the nuclear translocation of NFκBp65, exerting a therapeutic effect comparable to that of MSCs [70]. In an inflammation-induced infant brain injury model, intravenous injection of MSC-EVs effectively reduced microgliosis and prevented reactive astrocyte proliferation, thus promoting restoration of white matter microstructure [71]. Moreover, miR-124-3p enriched in neuronal EVs can inhibit the polarization of M1 microglia and A1 astrocytes, by activating PI3K/AKT to inhibit the NF-κB signaling cascade [72].

### Preventing blood vessel loss

Extensive blood vessel loss usually occurs following CNS injury, resulting in ischemia and hypoxia of the injured region. Preventing blood vessel loss and promoting regeneration enhances functional recovery after injury. In an SCI mouse model, microglia-derived exosomes protected spinal microvascular endothelial cells from the toxic effects of high oxidative stress and regulated angiogenesis after injury. The molecular mechanism underlying these effects is activation of the keap1/Nrf2/HO-1 signaling pathway through exosomes, which reduces the reactive oxygen species level in mice SCI lesions and promotes endothelial cell survival, as well as new blood vessel formation [73]. Another study showed that M2 microglia-derived exosomes activated Wnt/β-catenin signaling by inhibiting the ubiquitination of OTULIN protein and triggered the expression of angiogenesis-related genes in spinal cord microvascular endothelial cells (SCMECs) to mediate angiogenic effects [74].

### Maintaining BBB/BSCB integrity

The BBB/BSCB is a physical barrier that maintains normal CNS homeostasis. BBB/BSCB destruction leads to the infiltration of a large number of cells, imbalance in the internal environment, and secondary damage. Therefore, maintaining BBB/BSCB integrity is a major goal in CNS injury repair.

Intravenous injection of BMSC-EVs can stabilize the BSCB and enhance functional recovery after SCI. A previous study found that administered BMSC-EVs did not flow directly to the injured site; instead, they were taken up by M2 macrophages, resulting in the upregulation of transforming growth factor-beta (TGF-β), TGF-β receptors, and tight junction proteins, which in turn reduced BSCB permeability and promoted functional recovery [75]. Pericytes are essential components of the neurovascular unit and play an important role in maintaining BSCB structural integrity. BMSC-EVs reduced pericyte migration after injury and enhanced BSCB integrity by downregulating NF-κB p65 signaling [76]. Furthermore, the administration of pericyte-derived exosomes to SCI mice was associated with improved microcirculation, protection of BSCB, and reduced edema [77]. Recently, tissue inhibitors of matrix metalloproteinase 2 (TIMP2) were shown to attenuate the decrease in cellular junction proteins by inhibiting the matrix metalloproteinase (MMP) pathway. TIMP2 knockout in BMSC-EVs significantly reduced the efficacy of BMSC-EVs, indicating that the TIMP2/MMP signaling pathway is also involved in the ability of BMSC-EVs to maintain BSCB integrity and enhance functional recovery after SCI [78].

### Inhibiting neuronal apoptosis

Apoptosis refers to programmed cell death, which mainly occurs in the early stages of CNS [79]. Treatment with EVs at the early stage can successfully inhibit neural apoptosis and reduce the cascade response after injury.

Several studies have shown that EVs from different cellular sources play an important protective role in traumatic CNS injury by attenuating apoptosis. MSC-EVs reduced SCI-induced neuronal apoptosis by inhibiting the expression of PTEN/PDCD4 or regulating the apoptosis-related protein [80, 81]. Astrocyte-derived EVs can transfer NKILA (a long noncoding RNA) or gap junction alpha 1–20 k into neurons, thus decreasing neuronal apoptosis and improving functional recovery after TBI [82, 83]. Microglial exosomal miR-21 can regulate the expression of downstream apoptosis-related proteins and cleave caspase-3, caspase-9, Bcl-2, and Bax by activating the PTEN-Akt signaling pathway after TBI, thereby reducing neuronal apoptosis [84]. In an experimental SCI model, neural stem cell exosomes (NSC-EVs) alleviated neuronal apoptosis and neuroinflammation by inducing autophagy [85]. Following NSC-EV infusion, the expression levels of pro-apoptotic proteins and pro-inflammatory cytokines were reduced, whereas the expression of anti-apoptotic proteins was upregulated. However, the administration of autophagy inhibitor attenuated the beneficial effects of NSC-EVs, which suggests that NSC-EV treatment has the potential to promote functional recovery at an early SCI stage by promoting autophagy. Furthermore, MSC-EVs exert anti-apoptotic effects through activation of the Wnt/β-catenin signaling pathway [86].

### Regulation of autophagy

Autophagy plays a key role in physiological and pathological processes and is an important mediator of neuronal degeneration after injury. Neuronal exosomal miR-21-5p levels continue to increase from the acute to the chronic phase of TBI, and neuronal exosomes rich in miR-21-5p inhibit neuronal autophagy by targeting Rab11A activity, thereby attenuating autophagy-mediated neural damage *in vitro* [87]. The M2 BMDM-EVs target the mammalian target of rapamycin (mTOR) by delivering miR-421-3p to enhance neuronal autophagy and reduce apoptosis [88]. However, tail vein injection of miR-421-3p inhibitor showed that the in vivo protective effects of BMDM-EVs on the autophagy of neuronal cells were reversed. Furthermore, miR-124-3p-enriched microglial exosomes inhibited the family interacting protein of 200 kDa (FIP200)-mediated neuronal autophagy and promoted functional recovery after SCI [89]. Peripheral glial cells, i.e., Schwann cells, play an important role in the peripheral nervous system and CNS regeneration [90, 91]. Thus, Schwann cell-derived EVs may also be involved in CNS regeneration. The treatment of SCI rats with primary Schwann cell-derived exosomes can reduce apoptosis, promote axonal protection, and enhance motor functional recovery. The molecular mechanism underlying these effects involves the upregulation of autophagy by inhibition of the EGFR/Akt/mTOR signaling pathway [92].

### Promoting axon regeneration

The disruption of functional connectivity in the CNS leads to persistent dysfunction after injury; therefore, promoting axonal regeneration and plasticity is a major route to improving neural function.

Glial cells play an important role in promoting axonal growth. EVs from astrocytes and LPS-pre-activated astrocytes can activate the Hippo pathway, promote the expression of monopole spindle-binding protein 1 (MOB1), and reduce the level of Yes-associated protein (YAP), thereby facilitating neurite outgrowth and recovery after SCI [93]. The miR-151-3p is highly expressed in microglial exosomes and promotes axon regeneration in vitro and in vivo, which may involve downstream activation of the p53/p21/CDK1 signaling cascade [94]. In another study, activated microglial exosomal miR-5121 directly targeted the RGMa protein to promote axonal growth and synaptic recovery, which in turn improved the motor coordination of mice after TBI [95]. In addition, BMSC-derived exosomes enhanced the ability of macrophages to phagocytose myelin debris in vitro by promoting the expression of macrophage receptors with a collagenous structure (MARCO) in macrophages and created a regenerative microenvironment for axon regeneration [96].

## Applications of EVs

Because of their unique biological properties, EVs are increasingly being used as biomarkers and drug delivery systems for disease detection and prognostic prediction. Due to the poor targeting ability of native exosomes, surface-modified exosomes with additional functions have been developed to enable site-specific drug delivery, as discussed below.

### Biomarkers

Previous studies have demonstrated that exosomes can transport signaling molecules to nearby and distant cells, modulate recipient cell physiology, and participate in the pathogenesis and progression of various diseases [97]. Exosomes released by cells into the circulation and body fluids vary in terms of protein and RNA contents between healthy subjects and patients with different diseases [98–101]. This indicates that the release of the contents of EVs improves the disease microenvironment and may be a unique molecular feature of the disease itself.

RNA regulation is disrupted in various CNS pathological conditions, and changes in RNA expression levels may be indicative of certain diseases [102]. Using next-generation sequencing to analyze differences in serum exosomal miRNA profiles between sham and acute SCI rats, the expression of multiple miRNAs in serum exosomes was found to fluctuate. Among them, miR-130a-3p and miR-152-3p were upregulated, while miR-125b-5p was downregulated, suggesting that the miRNAs are good diagnostic markers of acute SCI [103, 104]. Brain-derived EV miRNA profiles also change after TBI. Experimental results showed that the expression levels of miR-21, miR-146, miR-7a, and miR-7b increased significantly, while the expression level of miR-212 decreased significantly, after injury [105].

Furthermore, analysis of proteins carried by EVs can effectively predict disease progression and injury severity. After TBI, the injured brain secreted EVs of different sizes, ranging between 99 and 104 nm, in control cerebrospinal fluid (CSF) samples, while the size decreased to 74–98 nm after trauma. In addition, the total quantity of EVs released into the CSF after injury was increased, and the ratio of these proteins was altered [106]. In another study, patients with a history of repetitive mild TBI (mTBI) showed higher exosomal and plasma levels of NfL (a neuronal marker of axonal injury). The elevated exosomal and plasma levels of NfL were associated with the postconcussive syndrome (PCS), posttraumatic stress disorder (PTSD), and depression, indicating that this exosomal protein can be used as a biomarker for the diagnosis of mTBI, PCS, PTSD, and depression [107].

Many studies exploring the use of exosome contents as biomarkers are underway. Although previous studies showed an association between EV biomarkers and neurological disorders, specificity and reliability were limited, which needs to be addressed in future studies [101]. Therefore, improved methods for purifying EVs, and the use of other clinical modalities (such as MRI) for functional validation, are necessary to increase the reliability of inspection tests [108].

### Drug delivery

Traditional medicines for neurological diseases often have limited efficacy and clinical applications due to their poor water solubility, rapid in vivo clearance, low biocompatibility, and poor cell permeability [4]. Exosomes combine the advantages of cells and nanotechnology and can pass through the BBB/BSCB. In recent years, exosomes have shown significant application potential as drug carriers in the treatment of neurological diseases.

To date, several approaches for exosome loading have been proposed and can be divided into two distinct strategies: cargo loading during biogenesis and cargo loading after isolation [109, 110]. Loading EVs during biogenesis requires blockade of the endogenous loading mechanisms of progenitor cells to generate EVs containing specific molecules, such as drug molecules and miRNAs [109, 111]. For example, BMSCs can be packed in their own EVs by co-incubating with paclitaxel, and the release of such EVs can exert anti-tumor effects [112]. Cargo loading after isolation is a more common strategy, and the earliest drug delivery systems load specific exogenous molecules into EVs [109, 113–115]. For example, miRNAs, superparamagnetic iron oxide nanoparticles (SPIONs), small-molecule chemotherapeutics, and bioactive macromolecules can be directly mixed with exosomes (rather than mother cells) by electroporation to obtain desired exosomes [116]. In addition to electroporation, engineered EVs, viral packaging mechanisms, and incubation have been used for EV molecular reprinting [117]. However, due to the selective sorting mechanism of parental cells for exosome components and limitations of transfection technology, unwanted cell-derived proteins/RNAs may be accidentally incorporated into EVs regardless of the loading method [118]. Therefore, the feasibility and efficiency of EV loading should be investigated further, and new techniques need to be considered to prevent the incorporation of unwanted molecules into the released vesicles [119].

EVs have also been used as carriers of low molecular weight drugs to treat diseases, which has achieved desirable results in mouse models of CNS injury. The nano-formulation of combined curcumin and embryonic stem cell exosomes effectively reduced infarct volume and edema in the injured area after cerebral ischemia and enhanced neurovascular recovery after ischemia–reperfusion injury [120]. In another preclinical experiment, exosomes derived from microglia treated with resveratrol were collected and injected into SCI mice; the exosomes enhanced the solubility of resveratrol and allowed stable entry thereof through the BBB to the damaged area. The PI3K/AKT signaling pathway was activated by resveratrol-containing exosomes, which showed higher neuronal survival and autophagy rates, while the level of apoptosis was reduced; this alleviated SCI more effectively than free resveratrol [121]. Although progress has been made in the development of EVs as drug delivery systems, no standard techniques or guidelines for the clinical use of EV therapy exist, and extensive preclinical and clinical studies are still required.

### Surface-functionalized EVs

Studies have demonstrated that EVs promote CNS recovery through their role as drug delivery systems. However, it is unclear how EVs can be safely and effectively delivered to the injured area. When exosomes are administered to animals, they preferentially accumulate in the kidneys and spleen and are eliminated through phagocytosis by the visceral system [118]. Only a small quantity of EVs reach the injury site. To improve the targeting efficiency of EVs, surface-functionalized EVs that carry surface-modifying molecules have been developed.

Currently available strategies for surface modification of EVs include genetic engineering, covalent modification, and non-covalent modification, among which covalent modification (i.e., chemical modification) is the most widely used [20, 122]. Genetic engineering combines the gene sequences of guide proteins or peptides with those of selected EV membrane proteins and is only suitable for targeting genetically encoded motifs [123]. As early as 2011, researchers were able to target cortical neural progenitor cells by fusing dendritic cell-derived EVs expressing Lamp2b with rabies virus glycoprotein (RVG) peptides, which effectively promoted neurogenesis after ischemia through the involvement of exosomal miRNA-124 [124]. The chemical modification requires the modification of exosome surface proteins through coupling reactions for assembly into new synthetic ligands; click chemistry is the most commonly used method in this strategy. In a mouse model of transient middle cerebral artery occlusion, the c(RGDyK) peptide was conjugated to the exosome surface via a biorthogonal click reaction, and this exosome could target cerebral ischemia when intravenously injected. Further loading of curcumin on cRGD-Exo can significantly inhibit the inflammatory response and apoptosis in the lesion area, indicating the targeting ability of this engineered exosome [125]. In another preclinical experiment of cerebral ischemia, the researchers designed an exosome with RVG peptides on the surface to target neurons and loaded nerve growth factor (NGF), which showed that the surface-modified exosomes maintain high NGF stability, thereby prolonging the time that they remain effective in the body [126]. Surface-modified functionalized exosomes have also been used to target gliomas. In a previous study, myelin-1-targeting peptides (RGERPPR, RGE) were conjugated to exosome membranes using click chemistry and loaded with SPIONs and curcumin; glioma-targeted exosomes with diagnostic and therapeutic functions were obtained [127].

By incorporating targeting moieties in the exosome surface, exosomes can promote targeted delivery of EVs to the injury site, which improves treatment efficiency and eliminates off-target side effects of the encapsulated therapeutic cargo.

## Conclusion

In conclusion, the treatment of traumatic CNS disorders is challenging, and there are no effective strategies to restore the lost function. The adverse reactions associated with cell transplantation limit its clinical application. In recent years, EVs have been studied as a possible alternative to cell transplantation for the treatment of CNS diseases. The EVs secreted by cells have similar functions to the parent cell and interact with the target cell to mediate intercellular communication, thereby avoiding the risks associated with cell transplantation while maintaining the therapeutic effect and potential.

The mechanisms by which EVs play a beneficial role in neurological disease recovery have been explored in different studies, which have provided a good basis for the development of clinical treatments. In addition, EVs have been used as biomarkers and drug delivery vehicles for various diseases, for early diagnosis, monitoring, and determination of the treatment response. However, their reliability needs to be confirmed by technical studies and through comparison with other indicators of clinical diagnosis [128]. Surface-functionalized exosomes improve the targeting ability and treatment efficiency of EVs as natural carriers and represent an emerging research area in drug delivery systems [129]. Furthermore, EVs show significant potential for use in CNS diseases, but many problems remain in terms of clinical translation. Therefore, additional preclinical research is needed before the EVs can be used clinically.

## Data Availability

Not applicable.

## Abbreviations

CNS: Central nervous system
EVs: Extracellular vesicles
TBI: Traumatic brain injury
SCI: Spinal cord injury (SCI)
MSC: Mesenchymal stem cell
BMSCs: Bone marrow-derived MSCs
BBB: Blood–brain barrier
BSCB: Blood–spinal cord barrier
miRNAs: MicroRNAs
MVBs: Multivesicular bodies
rRNA: Ribosomal RNA
hUC-MSCs: Human umbilical cord MSCs
LPS: Lipopolysaccharide
PrP: Prion protein
BMDMs: Bone marrow-derived macrophages
NLRP3: Nucleotide-binding domain-like receptor protein 3
EF: Epidural fat
SCMECs: Spinal cord microvascular endothelial cells
TGF-β: Transforming growth factor-beta
TIMP2: Tissue inhibitors of matrix metalloproteinase 2
MMP: Matrix metalloproteinase
NSC-EVs: Neural stem cell exosomes
mTOR: Mammalian target of rapamycin
FIP200: Family interacting protein of 200 kDa
MOB1: Monopole spindle-binding protein 1
YAP: Yes-associated protein
MARCO: Macrophage receptors with a collagenous structure
CSF: Cerebrospinal fluid
mTBI: Mild TBI
PCS: Postconcussive syndrome
PTSD: Posttraumatic stress disorder
SPIONs: Superparamagnetic iron oxide nanoparticles
RVG: Rabies virus glycoprotein
